# Stereotactic Body Radiotherapy (SBRT) for Oligometastatic Lung Nodules: A Single Institution Series

**DOI:** 10.3389/fonc.2019.00334

**Published:** 2019-05-07

**Authors:** Rodney E. Wegner, Stephen Abel, Shaakir Hasan, Lana Y. Schumacher, Athanasios Colonias

**Affiliations:** ^1^Division of Radiation Oncology, Allegheny Health Network Cancer Institute, Pittsburgh, PA, United States; ^2^Department of Cardiothoracic Surgery, Allegheny Health Network, Pittsburgh, PA, United States

**Keywords:** SBRT, lung nodules, metastases, oligometastatic, SABR

## Abstract

**Aim:** Lung metastases from an extra-pulmonary origin occasionally present with a limited metastatic disease burden. In cases where metastatectomy is not feasible, stereotactic body radiation therapy (SBRT) represents a non-invasive, efficacious option. We report the outcomes of patients treated with lung SBRT in cases of limited metastatic disease.

**Methods:** We retrospectively reviewed outcomes in 44 patients with 50 lung nodules from various extra-pulmonary malignancies treated with SBRT. Fifty percent of the patients were male and median age was 64. The median number of nodules was 1 and 90% of patients had oligometastatic disease. Thirty-four percent of patients had extra-thoracic disease.

**Results:** Fifty lung nodules were treated with SBRT in 44 patients. Median dose was 48 Gy in 5 fractions with a median biological effective dose (BED) of 100 Gy_10_. Follow-up imaging was available for review in 96% of nodules. Median follow-up was 17.5 months. One year local control was 82%. BED >72 Gy_10_ predicted improved local control (90 vs. 57% at 1 year). One year overall survival following SBRT was 66%. There was no difference in overall survival if patients had extra-thoracic disease.

**Conclusion:** Lung SBRT is a safe, effective tool for treatment of limited lung metastases. Dose selection remains important for local control.

## Introduction

Historically, lung metastases from an extrapulmonic origin signified widespread tumor dissemination and overall poor prognosis. However, a subset of patients will present with limited metastatic disease burden, with metastatic involvement of only a few anatomic sites (i.e. oliogmetastatic disease). Though systemic therapy remains the primary treatment modality in these cases, aggressive local therapy has been utilized with moderate success (1).

Surgical resection (i.e., metastatectomy) represents the preferred local treatment strategy when technically feasible (2, 3). Unfortunately, a subset of patients will not be operative candidates due to medical co-morbidities, anatomic limitations, or even patient refusal. In these cases of inoperable disease, alternative approaches are often utilized; with stereotactic body radiotherapy (SBRT) representing a non-invasive, efficacious option.

Lung SBRT emerged as a viable alternative to surgical resection for patients with medically inoperable non-small cell lung cancer (NSCLC) (4, 5). Results from early RTOG trials showed local control rates in the range of 90%, with limited toxicity when treating peripheral lesions < 5 cm in size (5). Given the favorable outcomes and toxicity profiles seen with lung SBRT in NSCLC, investigations assessing the role of SBRT in patients with limited metastatic disease burden confined to the lung were soon to follow (6–9). To that end, results of ongoing trials utilizing SBRT in the oligometastatic setting were recently presented showing improved overall and progression-free survival compared to standard of care, thus supporting the role of local ablative therapy in cases of limited systemic disease (10, 11). Herein, we present the results of a cohort with limited lung metastases treated with SBRT at our institution.

## Methods

We retrospectively reviewed the records of patients with known or suspected metastatic extra-pulmonary disease treated with SBRT between 2008 and 2017 in this institutional review board (IRB) approved study. All methods were carried out in accordance with relevant guidelines and regulations of the IRB affiliated with Allegheny Heath Network at Allegheny General Hospital. Patients having histologic confirmation of metastatic disease within the lung were included, as were, cases in which there was a high degree of clinical suspicion based upon previous clinical/pathological staging of the extra-pulmonary primary, history of metastatic disease, and/or radiographic enlargement of nodules over time. Patients were excluded if they presented with lung tumors exceeding 5 cm, or if they had a history of prior chest radiation. Patients were treated after review of their case and clinical characteristics in a multidisciplinary setting including medical oncology, thoracic surgery, diagnostic radiology, and radiation oncology. Patient characteristics are outlined in Table 1.

**Table 1:** Patient, disease, and treatment-related characteristics.

**Patient characteristics**
Age	64 years (38–86)
Males	22 (50%)
Females	22 (50%)
ECOG	
0	15 (34%)
1	24 (55%)
2	5 (11%)
**Disease characteristics**	
Number of lung nodules	50
Size	1.3 cm (0.4–3.8)
Pre-SBRT SUV	3.7 (0.6–12.2)
Extra-thoracic disease	14 (34%)
Oligometastasis (<5 sites)	39 (89%)
Primary Site	
Colorectal	22 (50%)
Breast	6 (13.5%)
Head and neck	6 (13.5%)
Endometrial	3 (7%)
Other^*^	7 (16%)
Location	
Upper	26 (59%)
Middle	2 (4.5%)
Lower	22 (50%)
**Treatment characteristics**
Dose	48 Gy in 5 Fx (36–54 Gy in 3–8 Fx)
Planning target volume	12.92 cc (3.3–103 cc)
Chemotherapy prior to lung SBRT	33 (75%)

*ECOG, Eastern Cooperative Oncology Group; SBRT, Stereotactic Body Radiotherapy; SUV, Standard uptake volume; Gy, Gray; Fx, Fractions; cc, cubic centimeter*.

* *Includes renal (n = 2), thyroid (n = 1), bladder (n = 1), Ewing's (n = 1), cholangiocarcinoma (n = 1)*.

**Figure 1:**
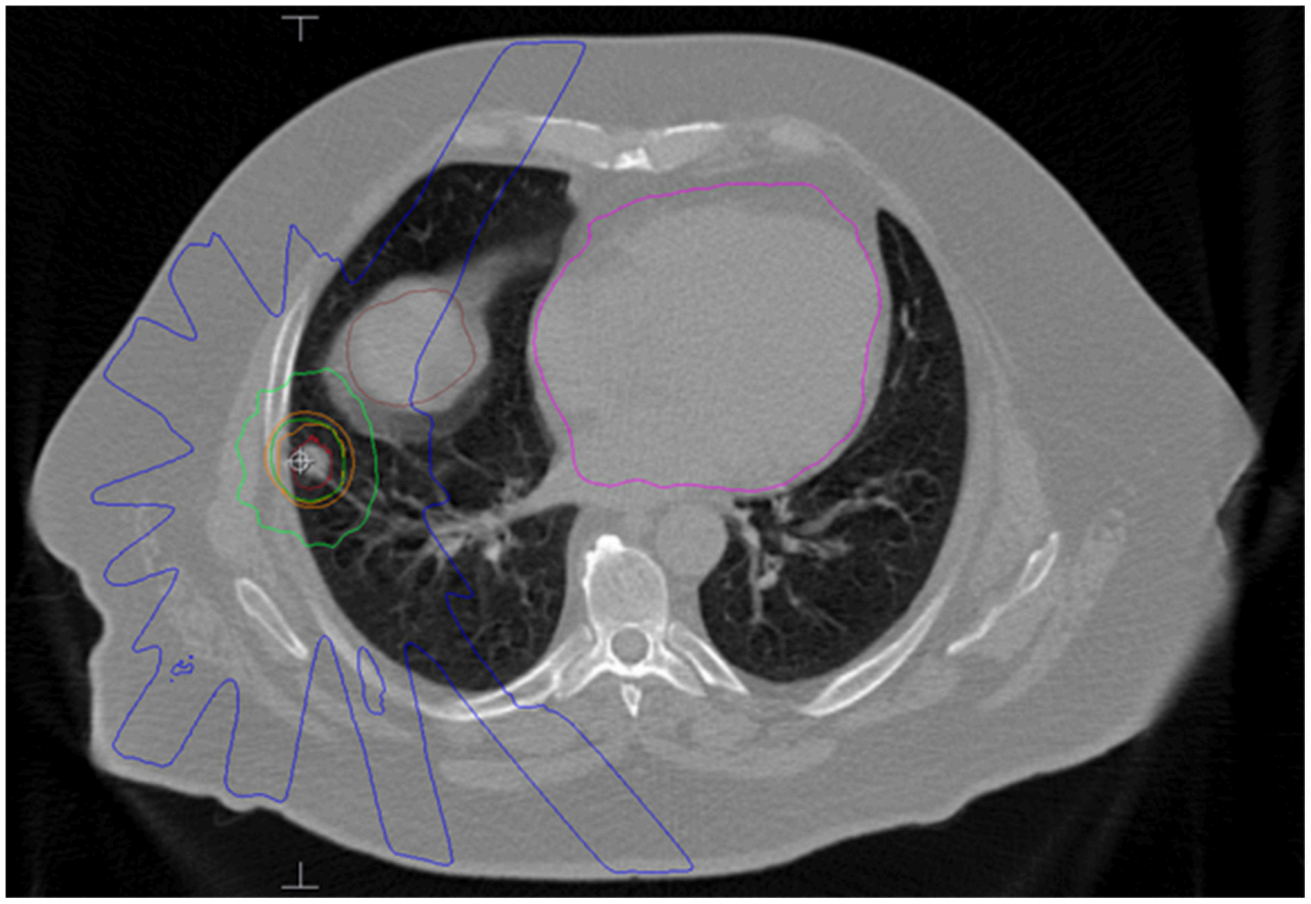
Axial slice of an SBRT plan for a patient with a single lung metastasis from a colonic adenocarcinoma. He received 50 Gy in 5 fractions. The red contour is the internal target volume (ITV) and the orange contour is the planning target volume (PTV). The dark green line is the 50 Gy isodose line. The light green line represents the 25 Gy isodose line and the blue line represents 10 Gy. The brown structure is the liver and the magenta structure is the heart.

SBRT was delivered in the outpatient setting using dose and fractionation schemes determined by the treating radiation oncologist. All patients underwent a 4-dimensional non-contrast chest CT using 1.5-3 mm slices for treatment planning simulation to account for respiratory motion. A gross tumor volume (GTV) was delineated on a free breathing scan and expanded on four expiratory and four inspiratory phases to generate an internal target volume (ITV) to account for intra-fractional motion. The planning target volume (PTV) expansion was 5 mm in all directions. Linear accelerator-based radiotherapy (without fiducial placement) was delivered via 8-12 coplanar 3D conformal beams with 6 MV photons. The median dose covering 95% of the PTV was consistent with the prescribed dose and a primary goal of treatment planning. Doses to surrounding organs at risk were reviewed and all attempts were made to meet constraints as outlined in the NCCN guidelines based on number of fractions (12). Daily megavoltage cone beam CT was used for image guidance to account for inter-fractional motion. Figure 1 shows a representative treatment plan.

After treatment, patients were typically followed with surveillance non-contrast chest CT or PET/CT at 3–6 month intervals for 1 year and every 6 months thereafter. PET/CT was obtained at the discretion of the treating medical or radiation oncologist, typically to determine response to prior therapy or restage the patient's disease. Response to treatment and local/distant control was assessed via RECIST criteria. Local failure was defined as an increase in the sum of the longest diameter of the target lesion by ≥ 20% from baseline. Distant failure was defined as any failure outside the treatment volume (including mediastinum, opposite lung, same lung). Patient and disease characteristics were reported (if available) and correlated with disease progression using univariate and multivariate analysis via Cox regression models (13). Survival, local control, distant control, and freedom from progression were all determined via Kaplan-Meier methodology using time from SBRT as the timeframe (14). All statistics were conducted via MedCalc Version 18.0 (Ostend, Belgium).

## Results

### Cohort

A total of 44 patients (22 males and 22 females) with 50 treated lung lesions from an extra-pulmonary primary were included in this study (Table 1). The median age was 65 years (range 38–86) with a median Eastern Cooperative Oncology Group (ECOG) performance status of 1 (range: 0–2). Thirteen patients (30%) had pathologic confirmation of the lung metastasis, with the remaining thirty-seven patients (70%) carrying a clinical diagnosis. Of note, 50% (*n* = 22) of patients had lung metastases from a colorectal origin. The median time from diagnosis of primary cancer to lung metastases was 26 months (range: 0–376). The median time to lung SBRT from primary diagnosis was 39.5 months (range: 4–377). Fifteen (34%) patients had extra-thoracic disease (metastatic disease to another organ outside the lungs/mediastinum) at time of SBRT and almost all (89%) patients had oligometastatic disease (defined here as 5 or fewer sites of metastasis). Seventy-five percent of patients had systemic therapy, typically chemotherapy, prior to receipt of lung SBRT. No patients had concurrent systemic therapy with SBRT. Ninety-three percent of patients went on to additional systemic therapy after SBRT. Twenty-seven (61%) patients had a pretreatment PET/CT, with median SUV in the treated nodule of 3.7 (0.6–12.2). The median number of nodules treated was 1 (range: 1–3). The median SBRT dose was 48 Gy in 5 fractions, ranging from 36 Gy to 54 Gy in 3 to 8 fractions with corresponding biologic equivalent dose (BED) range of 60 Gy_10_-105.6 Gy_10_. The median PTV volume was 12.92 cc (range: 3.3–103 cc). Of note, one single lesion received 8 fractions due to central location.

### Local Control

**Figure 2:**
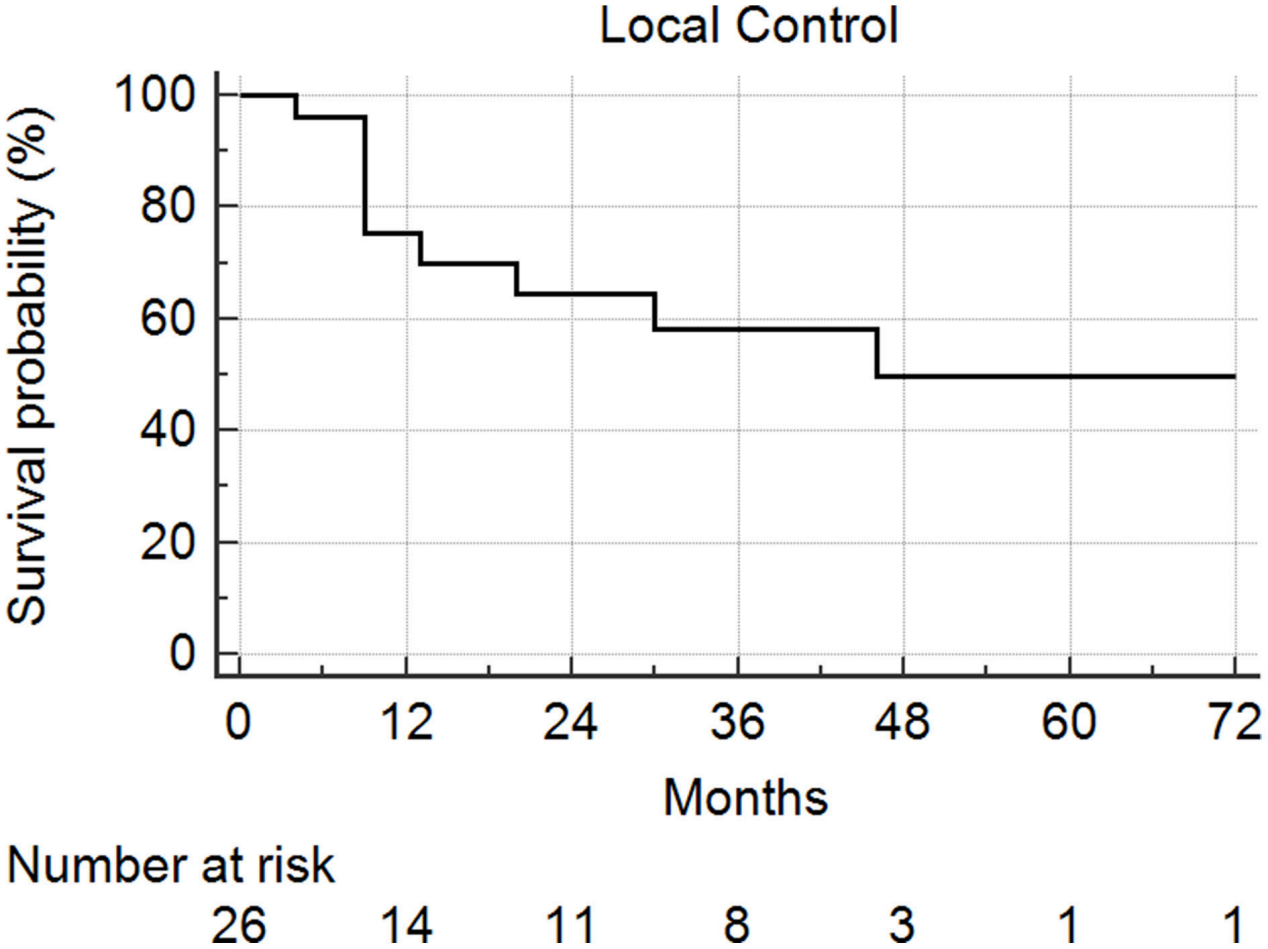
Local control following SBRT for lung metastases. Local control at 1 and 2 years was 82 and 74%, respectively.

**Figure 3:**
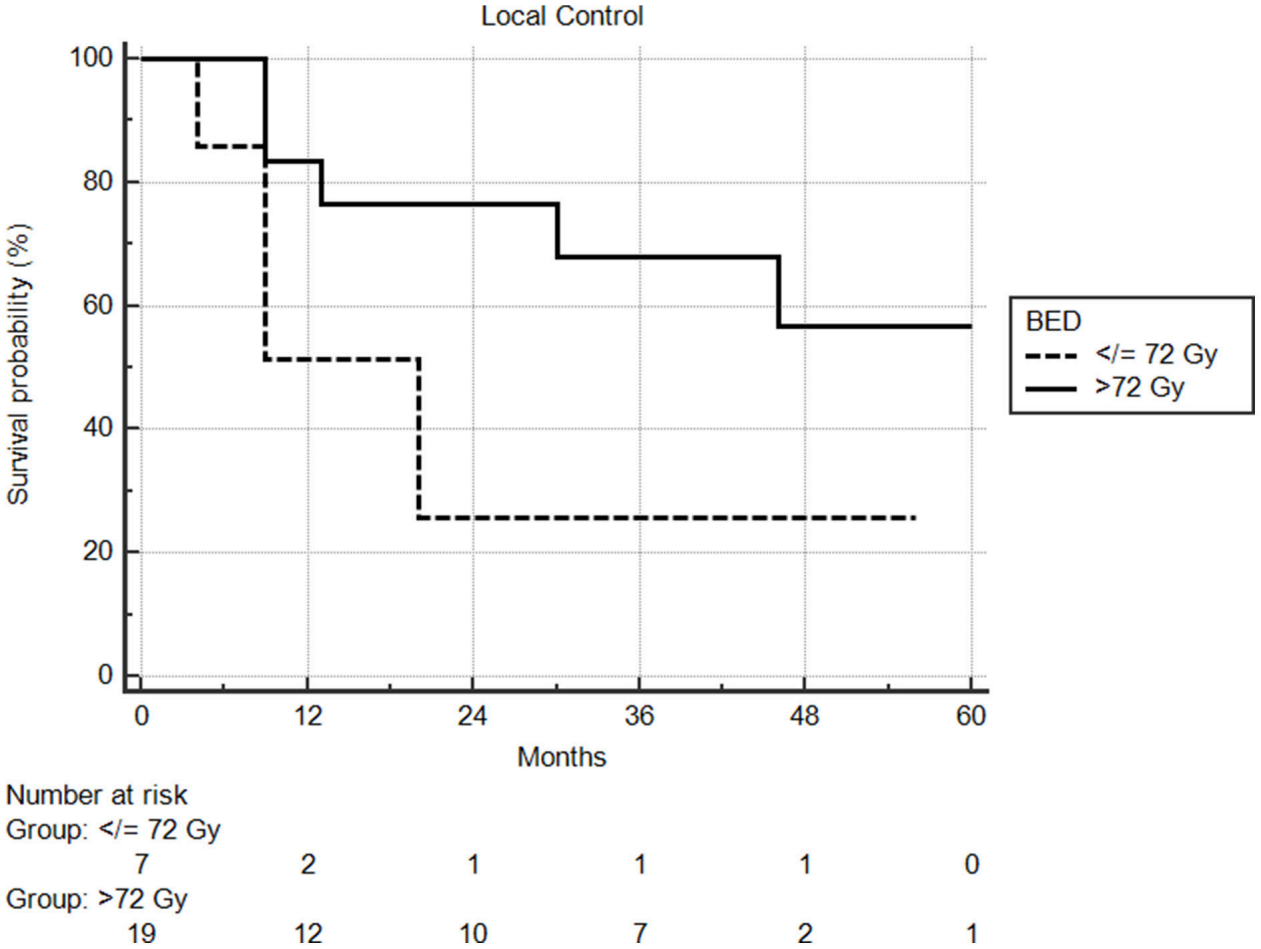
Local control by BED using a cutoff of 72 Gy. One year local control was 90% compared to 57%, in favor of higher biologic dose.

**Figure 4:**
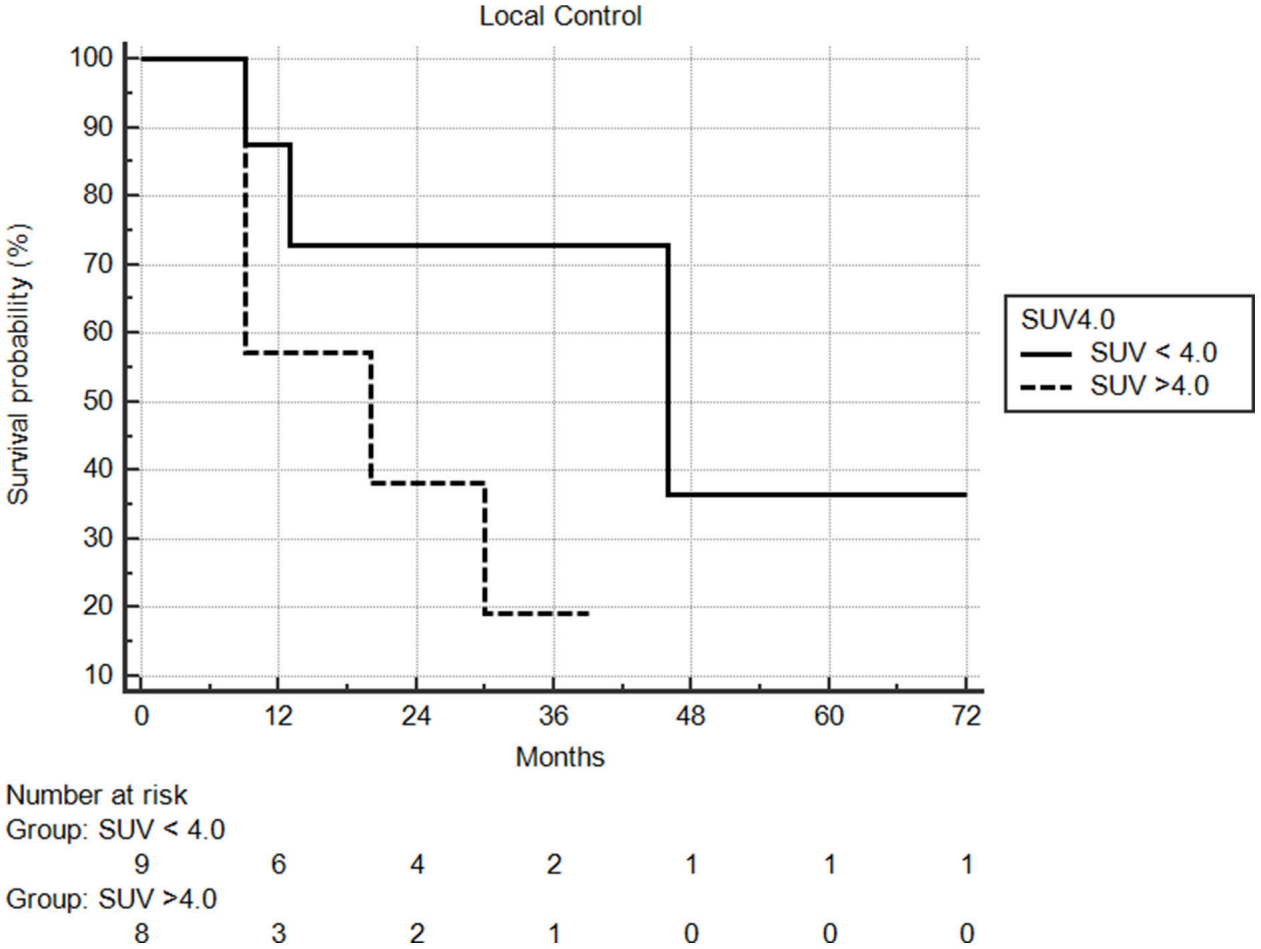
Local control based on pretreatment SUV. Local control at one year was 92% compared to 67%, in favor of lesions with less avidity.

Median follow-up from SBRT was 17.5 months (range: 1–68) and median follow-up from primary diagnosis was 56.5 months (range: 9–409). Follow-up imaging was available for 48 of 50 nodules (96%), with a median number of follow-up scans of 4 (range: 1–14). Median local control was not reached; however 1 and 2 year local control rates were 82 and 74%, respectively (Figure 2). Notably, local control was not influenced by PTV volume, histology, or anatomic location likely due to the small sample size and heterogeneity of the cohort. However, BED ≥72 Gy_10_ did show a benefit in terms of local control (1 year local control rate: 90%) compared to those with a BED < 72 Gy_10_ (1 year local control rate: 57%) (Figure 3). For the 27 patients having a follow-up PET/CT, lesions with SUV > 4.0 were more likely to have a local failure (33% at 1 year) compared to lesions with SUV ≤ 4.0 (8% at 1 year) (Figure 4).

### Survival

**Figure 5:**
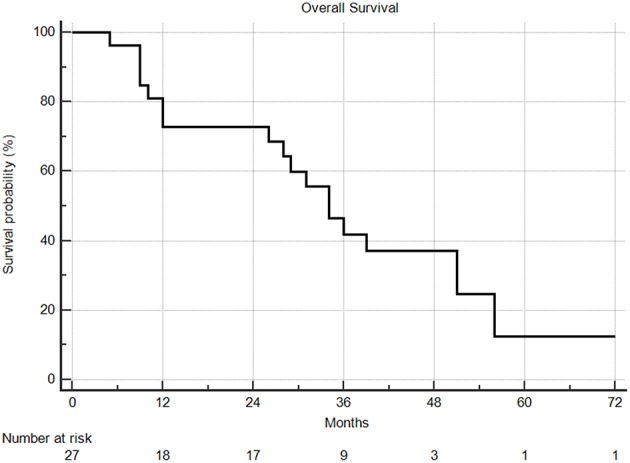
Overall survival from time of SBRT. Two and 5 year overall survival were 63% and 9%, respectively.

Median overall survival following SBRT was 29 months, with 1 and 2year overall survival rates of 66 and 63%, respectively (Figure 5). From the time of initial diagnosis, the median overall survival was 85 months. There was no difference in overall survival by extra-thoracic disease or oligometastatic status. PTV volume, dose, age, ECOG, tumor size, and fractionation did not predict for any differences in overall survival.

### Patterns of Failure

The median time to distant failure was 7 months, with a distant failure rate of 46 and 61% at 6 months and 1 year, respectively. No predictors were identified for distant failure. There was no acute or late grade 3 or higher toxicity noted in this patient population.

## Discussion

Lung metastases are relatively common and occur in 30–55% of cancer patients (1). Oftentimes, lung metastases are a harbinger of widely disseminated and essentially incurable disease. There are instances, however, where disease is truly limited, contemporarily referred to as oligometastasis (15). According to the concept, first described by Hellman and colleagues in 1995, the goal therein is to provide aggressive local therapy potentially rendering patients disease-free for a protracted interval. Criteria for defining oligometastatic disease varies by institution, protocol, and publication, keeping in mind additional factors such as total disease volume, genetics, histology, and location may also impact the outcome (16).

In terms of oligometastatic disease involving the lung, surgical resection is the current standard treatment. A group at Memorial Sloan Kettering reviewed outcomes from their institutional database including over 700 metastatectomies for lung metastases from sarcoma treated over a 25 year period (17). The median disease-free survival was relatively short at 6.8 months, but at 10 and 15 years 26% and 22% of patients were still alive, respectively. Another study from Denmark reviewed outcomes from various malignancies with limited pulmonary metastases treated surgically (18). This study included 178 patients with 256 surgical resections. At 5 years, survival for those with renal cell and colorectal cancers was 50%, while those with sarcoma and melanoma were 20–25% (15).

As previously stated, surgery is not always feasible for a variety of reasons. Generally, the accepted criteria for surgical resection include: adequate cardiopulmonary reserve, technical feasibility, control of primary tumor, and absence of extra-pulmonary disease (2, 3, 19). In situations where any or all of those criteria cannot be met, a slightly less invasive approach may be favored.

In those situations, SBRT represents a viable treatment option. A phase I/II trial from the University of Colorado enrolled 38 patients with 1–3 lung metastases from various primary sites. A 3 fraction SBRT regimen was utilized, with escalation of dose from 48 Gy to 60 Gy (20). With a median follow-up of 15 months, local control at 1 and 2 years was 100 and 96%, respectively. Toxicity was minimal with a single episode of symptomatic pneumonitis. A group from Rochester also has successfully demonstrated the efficacy of SBRT in treatment of oligometastatic disease (8). Results of this study were derived from a combination of two pilot studies which included all oligometastatic sites. Dose-fractionation was variable and dependent on anatomic location. The 2 year local control rate was 77%, with worse rates for larger tumors. In this particular study, lesions from gastrointestinal primaries tended to fair worse overall.

A group from Germany reported outcomes from a large multi-institutional series of 700 patients with medically inoperable lung metastases treated with SBRT (9). Patients in this study were treated using SBRT with a median fractional dose of 12.5 Gy (noting that they did include patients treated with >5 fractions). Median follow-up was over 1 year and local control at 2 years was 81%, with survival rate of 54%. They did note a 6.5% rate of pneumonitis, which was predicted by BED.

Within the past year, a few trials have presented exciting data showing improved outcomes utilizing SRS and SBRT in the oligometastatic patient. The first trial, SABR-COMET, was presented at ASTRO 2018 and enrolled close to 100 patients with various malignancies, defining oligometastatic state as up to 5 sites of metastatic disease (11). Therapeutic arms in that study were either standard of care or standard of care plus SBRT/SRS to sites of metastasis. The median overall survival was 41 months in the experimental arm compared to 28 months in the standard of care arm. Similarly, progression free survival was improved from 6 months to 12 months in favor of SBRT. A similar study, also presented in 2018, enrolled 49 patients all with metastatic NSCLC with no evidence of progression after initial systemic therapy. In this study patients were required to have 3 or less sites of metastasis (10). Another key difference was that consolidative therapy in this study was either surgery or SRS/SBRT, which was compared to ongoing standard maintenance therapy. With a median follow up of over 3 years the median overall survival was 41 months compared to 17 months in favor of local consolidative therapy (*p* = 0.017). Progression free survival was likewise improved from 4.4 months to 14.2 months (*p* = 0.014).The results of these two studies are exciting, showing meaningful improvement in important outcomes for this patient population.

Comparing the results of the current study, our results mirror those mentioned above with excellent local control of over 80% at one year. In addition, based on the various dose schemes employed we were able to show improved local control for doses with a BED_10_ > 72. This finding is concordant with previous reports in patients with NSCLC, in which, increased BED (i.e., >100 Gy_10_) was associated with improved local control and survival (21). This difference in local control emphasizes the importance of dose selection, even in the metastatic setting. Interestingly, our results showed inferior local control for lesions with an SUV >4.0, perhaps indicating radioresistance and a role for dose escalation. However, caution is advised when interpreting this result, as we did not have pretreatment PET/CT scans in all patients (61%). Additionally, of those with a pre-treatment PET, only 10 patients had an SUV>4.0. Another noteworthy finding relates to the disproportionate number of patients (i.e., 50%) in our cohort having pulmonary metastases from a colorectal primary. A previous meta-analysis suggested poorer local control in cases of pulmonary oligometastases from colorectal primaries possibility due to greater radioresistance (22). Nevertheless, 5 year overall survival in our series was similar to those mentioned above, with a rate of 64%, showing that excellent outcomes are attainable in appropriately selected patients. Furthermore, most of our patients (75%) had prior treatment with chemotherapy, which still remains the cornerstone of treatment in the metastatic setting. Comparable to previous investigations, SBRT was well tolerated in our patient population, with no reports of serious toxicity (Grade 3+).

The limitations of our study are those inherent to any retrospective series including selection bias. In addition, when dealing with patients with metastatic disease, distant failure and death from non-pulmonary causes are significant competing factors, which can perhaps skew local control results. This factor must be taken into consideration when considering results of studies completed using a similar patient population.

## Conclusion

Lung SBRT remains a viable treatment option for patients with limited metastatic disease in the lungs from extra-pulmonary primaries, with high rates of local control and minimal toxicity. Dose selection is important, with increased local control with higher BED_10_.

## Ethics Statement

All methods were carried out in accordance with relevant guidelines and regulations of the Allegheny Health Network Institutional Review Board (IRB) at Allegheny General Hospital and all subjects completed an informed consent document prior to treatment initiation.

## Author Contributions

RW: project conception, data collection, manuscript construction. SA: manuscript editing, manuscript submission. SH: manuscript editing, data collection, statistical analysis. LS and AC: project conception, manuscript editing.

### Conflict of Interest Statement

The authors declare that the research was conducted in the absence of any commercial or financial relationships that could be construed as a potential conflict of interest.
